# *Gallus gallus *NEU3 sialidase as model to study protein evolution mechanism based on rapid evolving loops

**DOI:** 10.1186/1471-2091-12-45

**Published:** 2011-08-23

**Authors:** Edoardo Giacopuzzi, Sergio Barlati, Augusto Preti, Bruno Venerando, Eugenio Monti, Giuseppe Borsani, Roberto Bresciani

**Affiliations:** 1 Department of Biomedical Sciences and Biotechnology, Unit of Biology and Genetics, University of Brescia, viale Europa 11, Brescia 25123, Italy; 2 Department of Biomedical Sciences and Biotechnology, Unit of Biochemistry and Clinical Chemistry, University of Brescia, viale Europa 11, Brescia 25123, Italy; 3 Department of Medical Chemistry, Biochemistry and Biotechnology, L.I.T.A., University of Milano, Via F.lli Cervi 93, Segrate 20090, Italy

**Keywords:** rapid evolving loops, sialidase, birds, comparative genomics, peptide tandem repeat

## Abstract

**Background:**

Large surface loops contained within compact protein structures and not involved in catalytic process have been proposed as preferred regions for protein family evolution. These loops are subjected to lower sequence constraints and can evolve rapidly in novel structural variants. A good model to study this hypothesis is represented by sialidase enzymes. Indeed, the structure of sialidases is a β-propeller composed by anti-parallel β-sheets connected by loops that suit well with the rapid evolving loop hypothesis. These features prompted us to extend our studies on this protein family in birds, to get insights on the evolution of this class of glycohydrolases.

**Results:**

*Gallus gallus (Gg) *genome contains one *NEU3 *gene encoding a protein with a unique 188 amino acid sequence mainly constituted by a peptide motif repeated six times in tandem with no homology with any other known protein sequence. The repeat region is located at the same position as the roughly 80 amino acid loop characteristic of mammalian NEU4. Based on molecular modeling, all these sequences represent a connecting loop between the first two highly conserved β-strands of the fifth blade of the sialidase β-propeller. Moreover this loop is highly variable in sequence and size in NEU3 sialidases from other vertebrates. Finally, we found that the general enzymatic properties and subcellular localization of Gg NEU3 are not influenced by the deletion of the repeat sequence.

**Conclusion:**

In this study we demonstrated that sialidase protein structure contains a surface loop, highly variable both in sequence and size, connecting two conserved β-sheets and emerging on the opposite site of the catalytic crevice. These data confirm that sialidase family can serve as suitable model for the study of the evolutionary process based on rapid evolving loops, which may had occurred in sialidases. Giving the peculiar organization of the loop region identified in Gg NEU3, this protein can be considered of particular interest in such evolutionary studies and to get deeper insights in sialidase evolution.

## Background

Large surface loops contained within compact protein structures and not involved in catalytic process have been proposed as preferred regions for evolution of different structural variants within a protein family [1,2]. Residues present in these loops are, on average, expected to be involved in fewer intra-molecular interactions [3] and the resulting lowered constraint on side-chain identity makes loop regions candidates for rapid sequence divergence and evolution. These loops can result in novel structural variants eventually leading to the acquisition of novel functions and/or protein domains.

Sialidases or neuraminidases (EC 3.2.1.18) are a family of glycohydrolytic enzymes that remove terminal sialic acid residues from various sialo-derivatives, such as glycoproteins, glycolipids and oligosaccharides [4]. In mammals four enzymes have been identified: the lysosomal sialidase NEU1, the soluble or cytosolic sialidase NEU2, the membrane-associated sialidases NEU3 and NEU4; each one with different substrate specificity [5].

The typical structure of sialidases is a β-propeller composed by four anti-parallel β-sheets organized in 6 blades which compose a compact and stable structure due to hydrogen bonds between β-sheets [6]. Such a structure also results in several loops connecting the various β-sheets and not involved in the catalytic process. Indeed, evidences collected so far from sialidases characterized in mammals [5], zebrafish [7], bacteria [8-10] and viruses [11-13] as well as from trypanosomal trans-sialidase [14] have already demonstrated the presence of large and highly variable loops regions between β-sheets. Taken together these evidences suggest that the sialidase protein family could effectively be studied to get deeper insights on the rapid evolving loop hypothesis mentioned above. Following this intriguing scenario, we focused on the state of sialidase genes in another lineage in which genomic data has recently become available: avians and particularly chicken. Chicken has been used as animal model in several research fields [15,16] and the study of bird genome is seen as a key to better understand vertebrate evolution. Efforts in avian genomics have led to the complete genome sequence of chicken in 2004 [17], and of turkey (*Meleagris gallopavo*) [18] and zebra finch (*Taeniopygia guttata*) in 2010 [19]. The analysis of the sialidase gene family in birds led us to the identification of a NEU3 protein with a long amino acid repeat region that appears to be specific for chicken and strictly related avian species, such as turkey. Here we describe the peculiar features of this region in *Gallus gallus *NEU3. Our data support the occurrence of rapid evolving loops in the sialidase protein family.

## Results

### Identification of sialidase genes in *Gallus gallus*

TBLASTN and BLAT searches conducted on chicken sequences using the four human sialidase proteins (NP_000425, NP_005374, NP_006647, NP_542779) as queries resulted in four chicken genes representing the putative orthologs of human *NEU1-4 *genes. The bioinformatic approach allowed us to recover the entire coding sequence of all chicken sialidases but NEU1, for which only one EST sequence is present in public sequence databases and no genomic sequence is available. The percentage of identity between human sialidases and the putative orthologous proteins in *Gallus gallus*, as obtained from BLASTP comparison, together with the localization of the corresponding genes are given in Table 1. The unusual dimension of Gg NEU3 protein prompted us to a more detailed characterization of this member of the gene family. Search in the Entrez Gene database for sequences annotated as *NEU3 *in *Gallus gallus *led to the identification of a mRNA (XM_428099.2) encoding a protein with high sequence identity to the middle and C-terminal region of human NEU3, but lacking the N-terminal portion. The identification of 27 chicken ESTs showing significant similarity to human NEU3 in a TBLASTN search allowed us to extend on the 5' side the XM_428099.2 sequence and to assemble a cDNA contig of 2421 bp (GQ365760). This sequence contains a 1908 bp ORF encoding a 636 amino acids protein with a calculated molecular mass of 69.8 kDa. A BLASTP search versus a non-redundant protein sequence database revealed that this novel protein shows the highest sequence identity to NEU3 of different vertebrates including *Homo sapiens *(51%) and *Mus musculus *(52%). According to the Chicken Gene Nomenclature Committee guidelines, the gene was named *NEU3*.

**Table 1:** Sialidase genes in *Gallus gallus*

Gene name	Dimension (bp)	Encoded peptide (aa)	Molecular weight (kDa)	Identity (%)	Loop region (aa)	Gene locus	Accession numbers
*Gg NEU1*	256	85	n.d.	58	n.d.	n.d.	BG710400 (EST) protein sequence manually translated from EST

*Gg NEU2*	1380	379	42.7	57	14	Chr9	XM_001231584 (mRNA) XP_001231585 (protein)

*Gg NEU3*	2124	556	69.8	53	204	Chr1	XM_428099 (mRNA) XP_428099 (protein)

*Gg NEU4*	1410	470*	51.3	50	56	Chr9	XR_027015 (mRNA)*

Essential data of the 4 putative orthologs of human sialidase genes identified in *Gallus gallus *based on a bioinformatic approach

n.d. = not determined; * = protein sequence translated from XR_027015 after mRNA sequence correction based on comparison with ESTs sequences

### Characterization of the *NEU3 *gene in *Gallus gallus*

**Figure 1:**
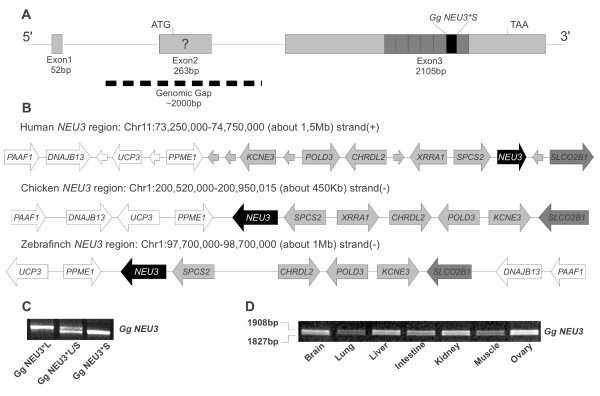
***Gg NEU3 *gene structure and chromosomal localization**. (A) Schematic representation of the putative structure of *Gg NEU3 *gene based on BLAT alignment of predicted *Gg NEU3 *mRNA with chicken genome. Exons (1-3) are represented by gray boxes and their length (bp) is indicated below. The repeat region is indicated as a dark grey area in Exon 3. The black area marked as Gg NEU3*S indicates the 81 bp region lacking in Gg NEU3 short allelic form. The question mark on Exon 2 indicates that its exact location could not be determined due to the presence of a gap (dotted bar) in the genomic sequence. (B) Schematic representation of the genomic region surrounding the *Gg NEU3 *gene on chicken Chr1, compared to the corresponding regions surrounding *NEU3 *on human Chr11 and on zebra finch Chr1. Arrows indicate the 5'-3' orientation of gene transcripts. Small arrows indicate human genes apparently not present in chicken and zebra finch. *NEU3 *genes are indicated as black arrows; white and light gray arrows indicate blocks of genes showing conserved syntheny; dark gray arrows indicate the intervening *SLCO2B1 *gene. (C) PCR amplification performed on genomic DNA from different specimens showing the presence of the two allelic forms in homozygous (Gg NEU3*L and Gg NEU3*S) as well as heterozygous state (Gg NEU3*L/S). (D) Reverse transcription mediated PCR with *Gg NEU3*_F and *Gg NEU3*_Ex_R oligonucleotides carried out on different tissues from the same chicken specimen. Products size is indicated on the left.

The *Gg NEU3 *gene is located in a 5609 bp region on chicken chromosome 1 and, like its human counterpart, it is likely organized in 3 exons (Figure 1A). We also analyzed the genomic region surrounding *NEU3 *in chicken and in man (Figure 1B). Conservation of synteny was observed between the two species with the exception of *PPME1, UCP3, DNAJB13 *and *PAAF1 *genes which in *Homo sapiens *are located more distantly from *NEU3 *(approximately 1 Mb) than in their chicken counterparts. As expected, conserved synteny around the *NEU3 *locus is observed also in zebra finch (*Taeniopygia guttata*). PCR amplification on chicken gDNA (see Methods section) resulted in two different products, supporting the presence of two allelic isoforms of the gene which we named NEU3*L (1908 bp ORF, GQ365760) and NEU3*S (1827 bp ORF, GQ365761) (Figure 1A and 1C), the latter encoding a polypeptide lacking 27 amino acids belonging to a tandem repeat region described in detail below. As the NEU3*L form was the one with a greatest EST support (data not shown), all further investigations were conducted on this variant. The presence of the two *NEU3 *polymorphic variants in the chicken population was investigated by PCR amplification of genomic DNA extracted from 10 different individuals. Our results demonstrated the presence of chickens homozygous for both alleles, as well as individuals that are heterozygous. An example of each genotype is shown in Figure 1C.

RT-PCR amplification with exon-spanning primers performed as described in methods revealed detectable transcripts in all tissue samples, in agreement with expression information available in BioGPS for the *NEU3 *gene in human and mouse tissues, with both allelic variants expressed at a comparable level (Figure 1D).

### NEU3 protein analysis in *Gallus gallus *and in other avian species

**Figure 2:**
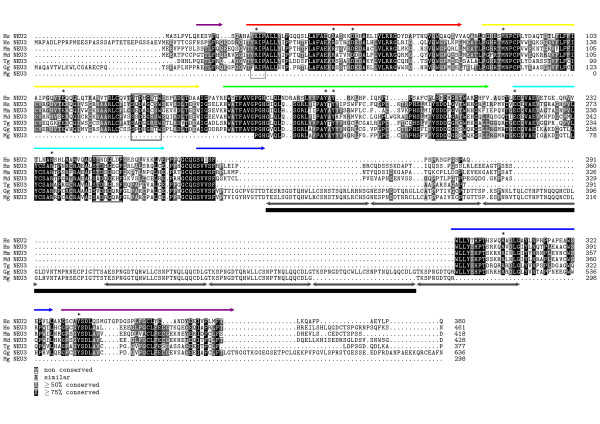
**Multiple sequence alignment of Gg NEU3 with other vertebrate membrane-bound sialidases and Hs NEU2**. Multiple alignment of the amino acid sequence of chicken NEU3 (Gg NEU3, GQ365760) with human sialidase NEU2 (Hs NEU2, NP_005374) and NEU3 from human (Hs NEU3, NP_006647), mouse (Mm NEU3, NP_057929), opossum (Md NEU3, XP_001366978), zebra finch (Tg NEU3, XP_002187487) and turkey (Mg NEU3, GU828006) performed with ClustalW2. Putative residues involved in the active site formation are indicated by *; (Y/F)RIP motif and Asp-boxes are framed with gray and black lines, respectively. The expanded region containing the repeats in chicken and turkey NEU3 is indicated with gray double arrows below the alignment, each one representing one of the 7 repeated units. The black bar indicates the region deleted in Gg NEU3-Del mutated protein. The 6 colored arrows above the alignment indicate the blades that compose the predicted Gg NEU3 β-propeller. The same color code is used in the 3D model in Figure 4.

**Figure 3:**
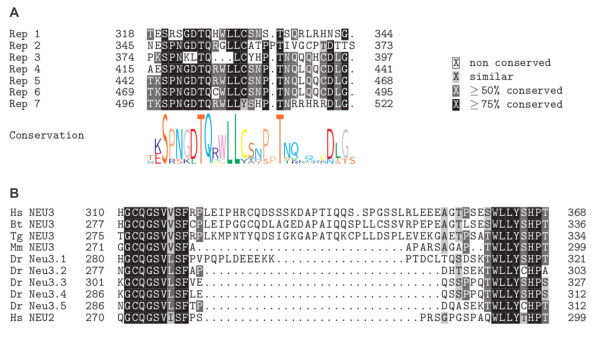
**Multiple sequence alignment of the Gg NEU3 repeat units and of the NEU3 variable length region in different species**. (A) Multiple sequence alignment of the 7 Gg NEU3 repeat units (Rep1-7). The positional information content according to the Logo analysis is shown at the bottom of the alignment. (B) Multiple sequence alignment of the NEU3 variable length region from different vertebrates located between the highly conserved GCQGSV(L/V/I)SF and WLLY motifs. NEU3 polypeptide sequences from *Homo sapiens *(Hs), *Bos taurus *(Bt), *Mus musculus *(Mm), *Taeniopygia guttata *(Tg), *Danio rerio *(Dr) and Hs NEU2 were aligned. The 188 amino acid-long sequence of Gg NEU3 containing the tandem repeats is located in this region but is not shown in the alignment due to its length. The alignments have been carried out using ClustalW2.

A multiple sequence alignment of Gg NEU3 with human NEU2 and NEU3, as well as with NEU3 from other vertebrates is shown in Figure 2. Computational analysis of Gg NEU3 performed with MotifScan and Pfam revealed the presence of the typical sialidase signatures: a (Y/F)RIP motif at residues 43-46, and three canonical Asp boxes [consensus SXDXGXX(W/F)] at residues 149-156, 222-230, 274-281. The protein is 636 amino acid long, with a predicted molecular weight of 69.8 kDa, a theoretical pI of 6.01 and a GRAVY value of -0,536 (slightly hydrophilic). The high level of sequence identity to human NEU2 (Hs NEU2), whose 3D structure has been solved [20] allowed the identification of amino acids possibly involved in the formation of the active site. All 11 residues involved in the coordination of the competitive inhibitor DANA in the active site of Hs NEU2 resulted conserved in Gg NEU3. Interestingly, the multiple sequence alignment revealed the presence in Gg NEU3 of a polypeptide region (encompassing residues 318-505) absent in all other NEU3 sialidases characterized so far, and responsible for the higher molecular weight of this enzyme of avian origin (see Table 1). The analysis of Gg NEU3 sequence with the Radar software showed that the region between positions 318 and 522 contains 7 repeat units (Rep1-7), starting with the mostly conserved sequence T(K/E)SPNGDTQ and repeated in a complex pattern. The complete organization of the repeat region within the NEU3 polypeptide is shown in Figure 2, while the single repeat units are compared in Figure 3A. In Gg NEU3 this region is composed by two blocks of repeats (Rep1-3 and 4-7), separated by a stretch of 17 unrelated residues (residues 398-414). Rep1 is preceded by another stretch of 13 unrelated residues (residues 305-317). There are 3 almost identical motifs (Rep4, 5 and 6) of 27 residues, 2 motifs (Rep7 and 1) of identical size but slightly different from those previously mentioned, and 2 less similar motifs of 29 and 24 residues (Rep2 and 3, respectively). Although Rep1-6 are peculiar of Gg NEU3, the last 2/3 of Rep7 result conserved also in NEU3 proteins from other vertebrates as well as in mammalian NEU2 (Figure 2) and NEU4 (not shown).

To investigate the conservation of the repeat region among avian species we identified the putative *NEU3 *gene both in turkey, *Meleagris gallopavo (Mg)*, and zebra finch, *Taeniopygia guttata (Tg)*. Figure 2 shows a portion of the Mg NEU3 polypeptide, identified as described in the Methods section, together with the putative Tg NEU3 amino acid sequence, retrieved from the NCBI database. Turkey NEU3 presents the tandem repeat region whose length corresponds to the one found in Gg NEU3*S chicken allele. Interestingly this region is absent in the Tg NEU3 polypeptyde, while the rest of the protein shows a high degree of sequence conservation (62% identity). As shown in Figure 3B, the chicken and turkey protein expansions are located in a region that is hypervariable in NEU3 proteins of mammals, birds and teleosts. This hypervariable region is included between two highly conserved amino acid blocks and, while Gg NEU3 shows the largest insertion (188 residues), its length increases from lower vertebrates (roughly 10 to 20 amino acids long) to mammals NEU3s (about 40 amino acids long).

### Gg NEU3 protein structure prediction

**Figure 4:**
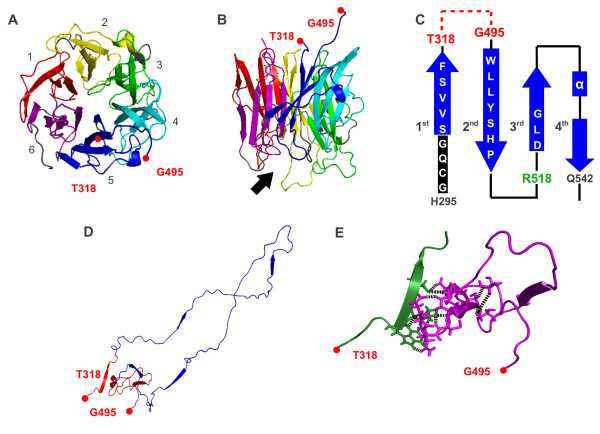
**Gg NEU3 structure prediction**. Top (A) and lateral (B) view of the 3D model of Gg NEU3 predicted using SWISS-Model. The numbers (1-6) in (A) indicate the six blades of the β-propeller and the black arrow in (B) indicates the catalytic crevice. The 6 blades of the β-barrel are shown with the same color code used in Figure 2. The first six non-conserved repeat units of the loop have been removed in this representation. First (Thr-318) and last (Gly-495) amino acids of the deleted region are indicated in red. (C) Topology diagram representing the arrangement of the fifth blade of the β-propeller. The blue arrows indicate β-strand and conserved amino acid blocks surrounding the repeat region are indicated with white letters. The repeat region between the Thr-318 and Gly-495 residues is represented by a red dotted line, whereas the other loop regions of the blade are represented by black lines. The catalytic residue Arg-518 is indicated in green. (D) *Ab initio *structure prediction of the loop region of Gg NEU3 (from Thr-318 to Gly-495). The loop-supporting region described in results is colored in red. The arrows indicate putative β-sheet regions within the structure as predicted by PSIPRED. (E) Detail of the support region of the Gg NEU3 loop. The first 12 residues of the loop are represented in green and the last 44 in magenta. Amino acids involved in hydrogen bonds or hydrophobic interactions are represented with sticks and linked with black dotted lines.

Prediction of Gg NEU3 structure (Figure 4A-B), based on its alignment with human sialidase NEU2 and performed using SWISS-Model, revealed, as expected, a high similarity with the NEU2 structure (1 snt). The predicted structure of the protein is the six blades β-propeller typical of sialidase [6], each blade being composed of four anti-parallel β-strands. The repeat region is located at the opposite side of the catalytic crevice and emerges as a long loop connecting two β-strands of the fifth blade of the β-propeller. This region is comprised between two highly conserved amino acid blocks, GCQGSV(V/I)SF (residues 296-304) and WLLY (residue 506-509) (Figure 2 and 4C). Based on Gg NEU3 structural model (Figure 4C), the highly conserved SVVSF block (residues 300-304) represents the first β-strand of the fifth blade of the β-propeller, followed by the expanded loop region containing the repeat units (residues 305-505). The next highly conserved region (residues 506-522) is part of the seventh repeat unit organized in: i) WLLYSHP (conserved residues 506-512), as second β-strand of the fifth blade of the propeller; ii) TNRRHRR (less conserved residues 513-519), as loop portion connecting the two anti-parallel β-strands and containing the essential catalytic residue Arg-518 and; iii) DLG (conserved residues 520-522), as first portion of the third β-strand of the same blade. The *ab initio *structure prediction on the repeat region peculiar of Gg NEU3 (Thr-318 to Gly-495), performed with ROSETTA and refined using I-Tasser, resulted in a long hairpin architecture with no particular secondary structure features (Figure 4D). Several hydrogen bonds and hydrophobic interactions occur between the first 12 and the last 44 residues of this region (in red in Figure 4D and detailed in Figure 4E). The 6 putative β-sheets predicted in the repeat region by PSIPRED are also indicated in Figure 4D.

### Expression and characterization of Gg NEU3 enzyme

**Figure 5:**
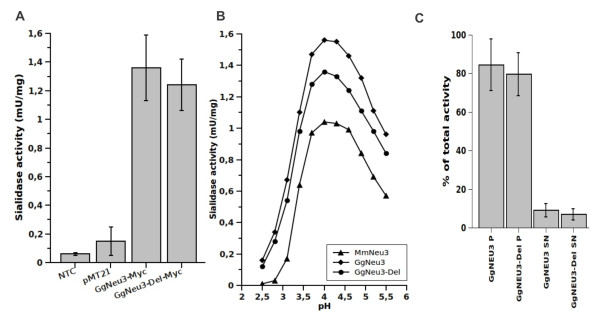
***Gg NEU3 *and *Gg NEU3-Del *expression in COS7 cells**. Sialidase activity measured with total cell lysate from COS7 cells transfected with pMT21-*Gg NEU3-*Myc, pMT21-*Gg NEU3-Del-*Myc, pMT21 vector alone or no DNA (NTC) using 4-MU-NeuAc as substrate. (A) Sialidase specific activity in the total cell lysate. The 95% C.I. error bars indicate variation in the observed activity. (B) Sialidase specific activity over the pH range 2.5-5.5. pcDNAI-*Mm NEU3-*HA transfected cells were used as control. (C) Repartition of sialidase activity in the supernatant (SN) and pelleted (P) fractions obtained after 100,000 ***g ***ultracentrifugation. Values in the bar graph are expressed as percentage of the activity detectable in total extracted material.

To demonstrate that the *Gg NEU3 *gene encodes for an active sialidase, the entire ORF was subcloned in the mammalian expression vector pMT21 and the resulting construct was used to transiently transfect COS7 cells. Crude homogenates from cells transfected with pMT21-*Gg NEU3- *Myc or pMT21 alone were tested for sialidase activity using the artificial substrate 4-MU-NeuAc. Transfection with pMT21-*Gg NEU3*-Myc resulted in a 9.1-fold increase in the enzymatic activity detected in the crude extract (Figure 5A). The enzyme showed a pH optimum at 4.0, similarly to bovine, human and mouse NEU3 enzymes [5] (Figure 5B). Fractionation by ultracentrifugation of crude extracts from transfected COS7 cells clearly demonstrated the association of Gg NEU3 with the particulate fraction, as about 85% of the activity detected in the homogenate was recovered in the pelleted material (Figure 5C). This is in agreement with data available for the human [21] and mouse counterparts [22].

**Figure 6:**
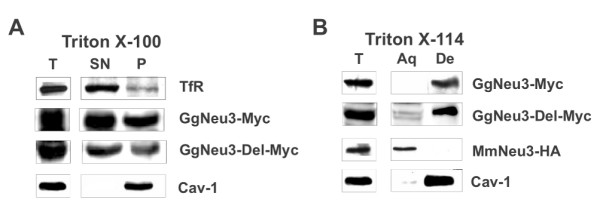
**Gg NEU3 and Gg NEU3-Del distribution after Triton X-100 extraction and after Triton X-114 treatment**. Detergent treatment were performed on total cell extracts derived from COS7 cells transfected with pMT21-*Gg NEU3*-Myc or pMT21*-Gg NEU3-Del-*Myc. (A) After extraction with Triton X-100 equal aliquots of the pelleted (P) and supernatant (SN) material were analyzed using anti-Myc, anti- Cav-1 and anti-TfR antibodies. Cav-1 was used as a DRM associated protein and TfR as typical membrane protein not associated to DRM. (B) After treatment with Triton X-114 identical aliquots of the aqueous [Aq] and detergent [De] phases were analyzed by Western-blot using anti-Myc, anti-HA and anti-Cav-1 antibodies. Mm NEU3-HA and Cav-1 were used as phase repartition markers. Total extract [T] analysis, prior to any treatment, is also given in both panel (A) and (B).

In order to study Gg NEU3 association to detergent resistant membranes (DRM), crude extracts were treated with Triton X-100 as described in methods. Immunoblotting analysis with anti-Myc antibodies revealed a partial recovery of Gg NEU3 in DRM fraction (Figure 6A), identified by the presence of Cav-1, a specific marker for these membrane domains [23]. This finding is in agreement with the results already obtained for human and mouse NEU3 [24,25]. Analysis of the hydrophilic/hydrophobic features of Gg NEU3 by aqueous-to-detergent repartition after Triton X-114 treatment revealed the almost complete segregation of Gg NEU3 in the detergent phase together with Cav-1, supporting a strong association with cellular membranes (Figure 6B). Interestingly, this behavior differs from that of Mm NEU3-HA, tested as a control protein, which segregated in the aqueous phase [26].

**Figure 7:**
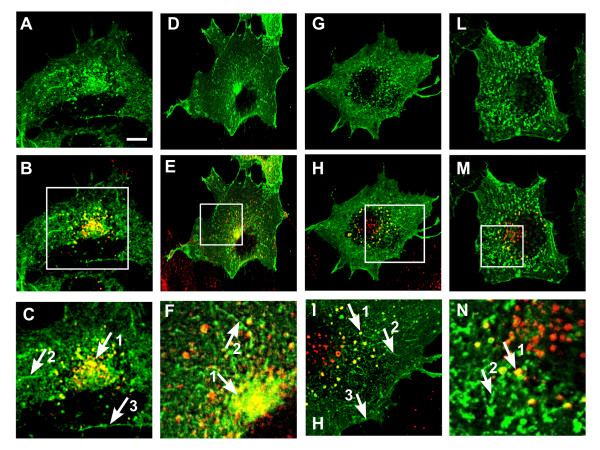
**Gg NEU3 and Gg NEU3-Del subcellular distribution in COS7 cells**. Cells were transfected with pMT21-*Gg NEU3-*Myc or pMT21*-Gg NEU3-Del-*Myc and subjected to immunofluorescence for co-localization experiments. Subcellular distribution of Gg NEU3-Myc (A, G) was related to TfR (red, merge in B) and EEA1 (red, merge in H). Subcellular distribution of Gg NEU3-Del-Myc (D, L) was also studied in relation to TfR (red, merge in E) and EEA1 (red, merge in M). Panels C, F, I, N represent a blow up of the squared regions in each B, E, H, M, respectively. Numbered white arrows evidence vesicular (1) and tubular (2) structures, as well as plasma membrane (3). Single confocal planes are shown. Scale bar: 10 μm

Immunofluorescence localization was studied in COS7 cells transiently transfected with pMT21-*Gg NEU3*-Myc at 36 h post transfection. Laser confocal microscopy analysis showed plasma membrane labelling together with intracellular tubular and vesicular structures, the latter being mainly concentrated in the juxtanuclear region of the cell (Figure 7A, G). These observations are in agreement with the localization of human and mouse NEU3, also performed in COS7 cells [21,22,26]. Co-localization experiments carried out using makers related to vesicular trafficking and protein recycling, such as TfR and EEA1, are illustrated in Figure 7 (panel B and H, detail C and I). Co-localization of Gg NEU3 with TfR and EEA1 indicate that, beside its localization at the plasma membrane, the protein is present also in early and recycling endosomal compartments, as already reported for Mm NEU3 [26].

### Gg NEU3 mutant protein properties

In order to evaluate the impact of the repeat region on the general properties of Gg NEU3 we carried out mutagenesis of the original protein as described in methods. This allowed us to generate a new protein of 458 amino acids (Gg NEU3-Del) with a predicted MW of 50.3 kDa, lacking 6 of the 7 units contained in the repeat region described above. In detail, we performed a 178 amino acids (531 bp) deletion from Thr-318 to Gly-495, leaving intact the seventh repeat unit and a 13 amino acids spacer (from Val-305 to Asp-317). The seventh repeat unit was maintained in the mutated construct since it contains fundamental structural elements and the enzyme catalytic residue Arg-518 (Figure 2). Overall Gg NEU3-Del and the wild-type protein showed superimposable results in the biochemical and cellular studies we performed. Indeed, the transfection with pMT21-*Gg NEU3-Del*-Myc resulted in a 8.3-fold increase of the enzymatic activity detected in the crude extract (Figure 5A) with a pH optimum at pH 4.0 (Figure 5B). Fractionation of crude extracts from transfected COS7 cells after ultracentrifugation showed that about 80% of the activity detected in the homogenate was recovered in the pelleted material (Figure 5C). The mutant protein repartition after treatments with Triton X-100 and Triton X-114 is also comparable with the results obtained with the wild-type protein (Figure 6). Finally, the subcellular localization in COS7 transfected cells showed no significant differences in the distribution of the wild-type and mutant protein (Figure 7).

## Discussion

The insertion of loop(s) of various lengths and not involved in catalytic activity, between two structurally conserved motifs, has been already observed in the Ras GTPases protein family [1] and could represent a mechanism for protein evolution and creation of novel protein domain(s) [1,2]. A preliminary survey of sialidase enzymes in vertebrates and mammals revealed the presence of a loop region which shows high sequence divergence and variable length in vertebrate NEU3 (from 9 amino acids in *Danio rerio *to 42 in *Homo sapiens*, see Figure 3B) and NEU4 (from 32 residues in *Danio rerio *to 80 residues in *Homo sapiens*).

Moreover as suggested by the sialidase characterization in *Danio rerio*, which posses 5 orthologs of human NEU3 gene but apparently no NEU2 ortholog [7], evolution of this protein family has been complex, probably following the increasing complexity of sialoconjugates from microorganisms to higher vertebrates [27,28]. To get better insight into evolution of the sialidase protein family, we decided to extend our studies to other vertebrates, based on the presence of rapid diverging loops in this class of enzymes. A bioinformatic analysis carried out on genomic and expressed sequences revealed that *Gallus gallus *genome, similarly to mammals, contains four genes that, based on sequence identity and synteny analysis, encode the putative orthologs of NEU1, NEU2, NEU3 and NEU4 proteins. The comparison of the primary structure of NEU3 from *Gallus gallus *with its human counterpart confirmed the presence in the former of an amino acid insertion larger than the one found in all NEU3 sialidases characterized so far. This large insertion may represent a rapid evolving loop and so a potentially important event for sialidase evolution. We thus decided to focus our interest on this member of the *Gallus gallus *sialidase family.

The *Gg NEU3 *gene is located on chicken chromosome 1 in a region of conserved synteny with chromosome 11q13.4 harboring *NEU3 *in *Homo sapiens*. While in both species the gene is flanked on one side by the *KCNE3, POLD3*, *CHRDL2*, *XRRA1 *and *SPCS2 *genes, an evolutionary rearrangement, probably an inversion of a 160 Kb region delimited by *NEU3 *and *KCNE3*, has separated *Gg NEU3 *from *PPME1 *in mammals. In chicken the *NEU3 *gene is apparently expressed in an ubiquitous manner, like its mammalian counterparts [29].

Primary structure analysis of the Gg NEU3 polypeptide revealed that, besides the high identity score with the human and mouse enzymes, all sialidase canonical amino acid signatures (i.e. (Y/F)RIP motif and Asp boxes) are present and conserved in topologically equivalent positions. The insertion identified in Gg NEU3 corresponds to a 188 amino acid region (Thr-318 - Arg-505) mainly constituted by a peptide motif repeated in tandem and organized in six plus one highly conserved repeat sequences (Rep1-6 plus Rep7). The analysis of 10 different chickens allowed us to identify a shorter allele of the gene characterized by the absence of a region encoding one of the repeats (presumably Rep6). These results demonstrate the presence of two alleles of *Gallus gallus NEU3 *gene.

The repeat cluster that characterizes Gg NEU3 may derive from complex duplication events originated from Rep7, as its last portion (residues 506-522) is conserved also in sialidases from other vertebrates. An almost complete identity is observed among Rep4, 5 and 6, whereas Rep1, 2 and 3 are more divergent, both in residue number and sequence, suggesting that duplication events occurred at different times during the evolution. Noteworthy, this repeat sequence is located between the evolutionary highly conserved sequence blocks GCQGSV(L/V/I)SF and WLLY. The analysis of the multiple sequence alignment in this region confirmed the presence of a loop of variable size in NEU3 vertebrate sialidases ranging from 9 residues, in the case of zebrafish Neu3.2-4, to 41-42 residues for the mammalian enzymes. Interestingly, an insertion of 80 and 78 residues is located in the same region in human [30] and mouse NEU4 [31], respectively.

Using the crystal structure of Hs NEU2 as template [20], a homology model of Gg NEU3 has been obtained, showing, as expected, the six-blade β-propeller architecture typical of sialidases [6,20]. The analysis of the model revealed that the repeat sequence found in chicken and turkey NEU3, as well as the stretch of about 80 residues found in mammalian NEU4s, are inserted at the opposite side of the active crevice. This region represents the loop connecting the highly conserved first and second antiparallel β-strands of the fifth blade of the β-propeller. The best model obtained for this loop region and based on *ab initio *structure prediction resulted in a long hairpin architecture with no particular secondary structure features (Figure 4D). Interestingly the peptide chain is stabilized by several hydrogen bonds and hydrophobic interactions occurring between the first 12 and the last 44 residues (Figure 4E). These interactions create a sort of support for the long loop and isolate it from the core of Gg NEU3 structure. This allows the exploration of new structural variants of the loop without impairing the protein biological activity. Along the hairpin chain 4 putative β-sheet regions have been also identified by secondary structure prediction and may contribute to stabilize this region.

In order to confirm that *Gg NEU3 *encodes for an active sialidase and the possible relevance of the loop, we expressed *Gg NEU3 *and *Gg NEU3-Del *cDNA in COS7, a cell line that has been used extensively for the biochemical characterization of sialidase enzymes [7,22,26,32]. Studies on transiently transfected cells showed for both proteins a significant increase in sialidase activity, with an acidic pH optimum, comparable subcellular localization and membrane association, in agreement with data on human and mouse NEU3 [21,22]. Interestingly Gg NEU3 and its deletion mutant revealed a peculiar hydrophobic feature when extracted with Triton X-114 compared to Mm NEU3 [26]. This feature probably does not depend on Gg NEU3 direct interaction with lipid bilayer, given its overall hydrophilic nature. A possible explanation for this behavior of the protein could be found in different interaction(s) of mouse and chicken NEU3 with other component(s) of the membrane. Overall, these data suggest that, in agreement with the rapid evolving loop hypothesis, the repeated region, at this stage of evolution, has apparently none or limited function(s) at least for the biochemical and cellular properties considered herein. It is intriguing to speculate about the fate of this loop region in sialidase evolution, particularly considering its increasing size, observed in NEU3 proteins of higher vertebrates, and the unique repeat organization observed in chicken and turkey.

In this perspective, the long amino acid stretch found in chicken and turkey NEU3, could represent an intermediate step in the evolutive process leading toward novel domain(s) relevant for the evolution of sialidases in this systematic group, as observed for lectin binding domain found in *Vibrio cholerae *[33]. Concerning the repetitive nature of the loop found in Gg NEU3 it should be noted that it does not represent a unique case among sialidases. Indeed, the C-terminal domain of the trans-sialidase (TS) from *Trypanosoma cruzi *contains a variable number of a tandemly repeated 12-amino acid-long unit, unrelated to the one found in Gg NEU3, termed SAPA (for Shed Acute- Phase Antigen) that is not required for TS activity [14,34]. Although some studies indicate that the repetitive amino acid motif stabilizes the catalytic activity of the enzyme in blood, its function remains largely unknown [34]. Moreover, our data demonstrate that the insertion of a large loop in this region of the enzyme does not impair the 3D organization of sialidase, indicating that this portion of the enzyme could be exploited as insertional spot for artificial modifications of the protein.

We demonstrate that also NEU3 from turkey contains the repeat region, whose length corresponds to the one of the chicken short allelic variant. This is not surprising considering that turkey is a domestic bird strictly related to chicken in the evolutionary tree of the *Galliformes *order. Besides chicken and turkey, the only other avian genome that has been sequenced belongs to the australian songbird zebra finch (*Passeriformes *order) [19]. We were surprised to discover that the zebra finch NEU3 completely lacks the repeat region, showing instead a short loop of 11 residues, similar in length to the one found in cytosolic sialidase Hs NEU2. This finding indicates that the repeat region is highly variable and is not a peculiar feature of all avian NEU3 sialidases, suggesting three evolutionary hypotheses. The repeat sequence has arisen: i) recently in the *Galliformes *order; ii) in the *Galloanserae *clade; or iii) in a common ancestor of both *Galloanserae *and *Neoaves*, and then has been lost in the latter at any point of the evolutionary tree leading to zebra finch [35]. By considering the first two hypothesis we can not exclude that the repeat region is present only in breeding birds as a consequence of the high genetic pressure and selection applied by man to these species [36]. The sequencing of additional avian genomes will allow to shed light on these evolutionary scenarios.

## Conclusion

In this study we demonstrated that sialidase NEU3 β-propeller contains a surface loop highly variable in both sequence and size connecting two conserved β-sheets and emerging on the opposite site of the catalytic crevice. In the case of chicken and turkey, both belonging to the *Galliformes *order, this loop is constituted by a number of repeats organized in tandem. The removal of the repeat region does not seems to alter the evaluated properties of the Gg NEU3 enzyme. Our data indicate sialidase family as good model to study the evolutionary process based on the rapid diverging loop hypothesis [1,2,37]. According to this theory, new random protein folding and architectures can emerge rapidly taking advantage of low constraint acting on surface loops at both sequence and structure level. When a loop develops into a useful or stable structure, it becomes subjected to positive selection and the new variant could be retained and eventually originate a new protein with specific features. This process may had occurred in sialidase protein family evolution leading to the membrane-bound enzyme forms, namely NEU3 and NEU4, present today in higher organisms. Giving the peculiar features of its loop region, *Gallus gallus *NEU3 could be of particular interest in such evolutionary studies. Additional work is needed to unravel a possible role of the repeat region in the avian biology.

## Methods

When not specified, standard molecular biology techniques were carried out as described by Sambrook et al [38]. Powder and reagents were from Sigma unless otherwise indicated. Oligonucleotide primer sequences for PCR amplification are reported in the corresponding sections with restriction sites underlined and, in case of degenerated primer sequences, the standard IUPAC nomenclature has been used.

### Identification of sialidase orthologous genes in *Gallus gallus *and bioinformatic analysis of chicken *NEU3*

The putative *Gallus gallus *sialidase genes (*Gg NEU1-4*) were identified using a "reciprocal BLAST" approach. Briefly, the human NEU1-4 protein sequences were used in a TBLASTN search to identify chicken ESTs or RefSeq mRNA similar to the query sequence. We similarly performed a BLAT search on the v2.1 draft assembly of *Gallus gallus *genomic sequence using the UCSC Genome Browser. The identified sequences were then aligned locally (using BLAST-P) and globally (using CLUSTALW2) with the corresponding human proteins to check for similarity and presence of the main sialidase signatures. Due to the peculiar feature identified with this approach in *Gg NEU3*, only this gene has been selected for further analysis. The *Gg NEU3 *ESTs were assembled using Invitrogen NTI Vector Suite v10.3 to obtain a 2421 bp *NEU3 *cDNA sequence contig. The Expasy translate tool [39] and the NCBI ORF Finder software [40] were used to predict the longest open reading frame present in this cDNA sequence. The Gg NEU3 predicted protein was aligned with NEU3 amino acid sequences from *Homo sapiens*, *Mus musculus*, *Takifugu rubripes *and other vertebrate sialidases using ClustalW v2.0. The *NEU3 *gene and amino acid sequence were subsequently analyzed using a set of public domain bioinformatic tools: RADAR [41] for the identification of internal repeat sequences; BioGPS [42] for the gene expression profile in different tissues and cell types; ProtParam [43], for physico-chemical properties; MotifScan [44] and Pfam [45], for conserved domains and functional motifs.

### Chicken tissue collection and sample preparation

All tissues used for *Gg NEU3 *gene cloning and expression analysis were from the same chicken specimen. The animal was sacrificed by neck translocation and small pieces from different organs (brain, lung, muscle, kidney, liver, intestine, and ovary) were rapidly obtained by manual dissection and immediately frozen in liquid nitrogen. Roughly 150 mg of every tissue sample were homogenized and RNA and genomic DNA were extracted using Trizol reagent (Invitrogen), following the manufacturer protocol. RNA samples were further purified using RNeasy mini kit (Qiagen) and the RNA integrity was evaluated using a Bioanalyzer 2100 (Agilent). Two μg of RNA sample were used for retrotranscription using the SuperScript III kit (Invitrogen) and oligo(dT) primers according to manufacturer's protocol. Nucleic acid concentrations were quantified using a ND-1000 spectrophotometer (Nanodrop Technologies).

### Identification of *Gg NEU3 *allelic variants and tissue expression

To validate the sequence of the predicted *NEU3 *gene in chicken, cDNA from liver of a single individual was used as template in a PCR amplification reaction with *Gg NEU3*_F (5'-GAGACACTGTTCCGACAGGA-3') and *Gg NEU3*_R (5'-CGTGTTGACATAGAGCCCCA-3') oligonucleotide primers designed to amplify the complete *Gg NEU3 *ORF. The PCR products were subcloned in the pMT21 plasmid vector. Automated DNA sequencing of different recombinant clones revealed the presence of two variants of the transcript: a long isoform with a 1908 bp ORF and a short one of 1827 bp.

To verify if these two variants are generated by alternative splicing or represent two different genomic alleles we conducted PCR amplification on genomic DNA extracted from the same individual, performed using *Gg NEU3*_Rep_F (5'-GTCCTGTAGGCACCACTGAC-3') and *Gg NEU3*_R primers belonging to exon 3 and flanking the tandem repeated region.

*Gg NEU3 *tissue expression was analyzed by semi-quantitative PCR on different cDNA sample prepared as described above using the *Gg NEU3*_F and *Gg NEU3*_Ex_R (5'- AGAAGATGAAGGAGTGCGGG-3') exon-spanning primers to avoid the effect of genomic DNA contamination. *HPRT *(hypoxanthine phosphoribosyltransferase 1) housekeeping gene was amplified using *Gg HPRT*_F (5'-AGGGCATGGGAGGACACCACA-3') and *Gg HPRT*_R (5'- GCACAACCCAACCAGGGGCA-3') primers to normalize for different efficiencies in RT-PCR reaction. PCR amplification was performed for both genes with the following parameters: initial denaturation at 95°C for 10 min followed by 27 amplification cycles (95°C for 30 s, 57°C for 30 s, 72°C for 1 min) and a final extension at 72°C for 5 min.

### Gg NEU3 structure prediction

To predict a structure for Gg NEU3, its amino acid sequence was first aligned with human NEU2, the only vertebrate sialidase whose 3D structure has been solved [20], and other mammalian sialidases using ClustalW v.2.0. The generated multiple alignment file was used in SWISS-Model [46] to superimpose the native NEU2 structure retrieved from RCSB protein data bank (1 snt) and predict 3D conformation of Gg NEU3. The predicted structure was visualized and analyzed using PyMol. The repeat region peculiar of Gg NEU3 (residues 318-495) was selected and removed from the whole protein structure. This portion of the protein, corresponding to a long loop, was submitted to ROSETTA for *ab initio *structure prediction, since no suitable template structure for 3D modeling of this sequence was present in public databases. The resulting predicted structure was refined using I-TASSER [47] to impose a constrain on the distance between Thr-318 and Gly-495 so that their positions fit in the predicted structure of the whole Gg NEU3 protein. The obtained final loop model was then assembled with the rest of the Gg NEU3 predicted structure to obtain a 3D model of the whole protein. Finally a simple secondary structure prediction was conducted on the repeat region primary sequence using PSIPRED [48].

### Degenerated oligonucleotide primed PCR (DOP-PCR) of turkey *NEU3 *sequence

We used degenerated oligonucleotide primed PCR (DOP-PCR) to investigate the presence of the repeat region in NEU3 from other avian species, namely turkey and peacock. First, the Gg NEU3 predicted protein sequence was aligned with human and mouse orthologs to identify most conserved regions. Based on this alignment, several degenerated oligonucleotide primers were designed in two highly conserved regions surrounding the repeated domain. After DOP-PCR optimization, a specific product of expected size was amplified only in turkey using Avian *NEU3*_F (5'-GGNTGYCARGGNWSNGTNGTNAGYTT-3') and Avian *NEU3*_R (5'- GTNGGRTGNGARTANARNARCCA-3') primers with the following reaction mixture: PCR GoldBuffer (Perkin Elmer), 600 nM of each primer, 5 mM MgCl_2_, 2.5 U TaqGold polymerase (Perkin Elmer), 0.4 mM dNTPs, 5% DMSO, 300 ng genomic DNA as template. After initial denaturation at 94°C for 9 min, the amplification reaction was performed for 45 cycles (94°C for 30 s, 48°C for 1 min, 72°C for 75 s) followed by a final extension step at 72°C for 5 min. We failed to amplify *NEU3-*related sequences from peacock genomic DNA. The turkey amplified product was gel purified using Gel Extraction kit (Qiagen) and cloned in pCR2.1-TOPO TA-vector (Invitrogen) using TOPO cloning kit for sequencing (Invitrogen) according to manufacturer protocol. Plasmid DNA was then extracted from positive colonies and sequence verified. A high level of sequence identity was detected with the corresponding region of *Gg NEU3 *gene, both at the DNA (91%) and at the protein level (84%). At a later stage of the project we were able to obtain a still unreleased sequence from the Turkey Genome Consortium (Virginia Bioinformatics Institute at Virginia Tech, USA) corresponding to a 1527 bp portion of the last exon of the *NEU3 *gene that partially overlaps with the DOP-PCR product we generated.

### Cloning of *Gg NEU3 *and expression in COS7 cells

The entire *Gg NEU3 *predicted ORF was amplified by PCR from chicken liver cDNA using the high fidelity TripleMaster PCR kit (Eppendorf), a forward primer with the EcoRI site (*Gg NEU3*_EcoRI, 5'-CGGAATTCATGGCTCAGGCTGTCACCTGGCT-3') and a reverse primer with the SalI site (*Gg NEU3*_SalI, 5'-ACGCGTCGACGATTAAAAGCCTCGCAACGCTGC-3'). pMT21-*Gg NEU3 *construct was generated by cloning the amplified insert in frame with the Myc epitope into pMT21 plasmid [49]. Plasmid DNA was extracted from positive colonies and the *Gg NEU3 *insert was sequence verified. The pMT21-*Gg NEU3-*Myc construct was used to transfect COS7 cells. Briefly, cells were plated in Petri dish (100 mm diameter, 400000 cells/dish) and transiently transfected with pMT21-*Gg NEU3-*Myc in serum-free medium (OptiMEM; Gibco-BRL) using 3 μg of DNA and 4.5 μl of FuGENE 6 (Roche). As positive control we transfected a pcDNAI-*Mm NEU3*-HA vector previously tested in our laboratory [26]. For indirect immunofluorescence COS7 cells were seeded onto 22 mm diameter glass coverslips (20000 cells/glass) and transfected with 1 μg of pMT21-*Gg NEU3-*Myc DNA, 1.5 μl of FuGENE 6. After 5 h in presence of the transfection medium, cells were washed with PBS and grown in DMEM supplemented with 10% FBS for 36 h when used for immunofluorescence or 48 h when used for enzymatic activity assay and Western-blot analysis.

### *Gg NEU3 *mutagenesis

A mutant construct lacking the repeat region (pMT21-*Gg NEU3-Del*) was obtained from the original pMT21-*Gg NEU3 *construct using the Quick-Change mutagenesis kit (Stratagene) according to the manufacturer protocols, optimized for long deletion as suggested in [50]. Briefly, we used two mutagenesis primers *Gg NEU3-Del*_F (5'- GGGTGTCCTGTAGGCACCACTGACACCAAGAGCCCCAATGGGGACAC-3') and *Gg NEU3- Del*_R (5'-GTGTCCCCATTGGGGCTCTTGGTGTCAGTGGTGCCTACAGGACACCC-3') for a PCR amplification with the following parameters: initial denaturation at 95°C for 3 min followed by the amplification reaction for 18 cycles (95°C for 30 s, 64°C for 1 min, 72°C for 14 min). After digestion for 2 h with DpnI enzyme, 5 μl of the resulting amplified product was used to transform XL-1 Blue *E.coli*. Positive colonies were screened by PCR with *Gg NEU3_*Rep_F and *Gg NEU3*_R primers flanking the repeated region to verify the correct size of the deletion and the construct was sequence verified. This construct (pMT21-*Gg NEU3-Del-*Myc) was transfected in COS7 cells as described in the above section.

### Sialidase activity assay

The enzymatic activity of Gg NEU3-Myc and Gg NEU3-Del-Myc was determined as previously described [26] using 4MU-NeuAc as substrate. One Unit of sialidase activity is defined as the liberation of 1 μmol of NeuAc/min at 37°C. To evaluate the association of the two proteins with cell membranes, the enzymatic activity was also measured on pelleted and soluble fractions obtained after centrifugation of the total cell extracts at 100,000 ***g ***for 1 h. In order to determine the optimum pH value for Gg NEU3-Myc and Gg NEU3-Del-Myc activity, total cell extracts were incubated in the same conditions as above using 11 different pH values, from 2.5 to 5.5.

### Antibodies

The following primary antibodies were used: for indirect immunofluorescence rabbit anti-Myc, mouse anti-EEA1 (Transduction Laboratories), mouse anti-TfR (Zymed), all diluted 1:200. For immunoblotting: mouse anti-Myc (obtained as cellular culture supernatant of commercial clone 9E10) undiluted, rabbit anti-HA, rabbit anti-Cav-1 (Santa Cruz) and mouse anti-TfR (Zymed), diluted 1:1000. For immunofluorescence experiments, donkey anti-rabbit Alexa 488 and donkey anti-mouse Alexa 555 (Molecular Probes) secondary antibodies were used, diluted 1:400. For immunoblotting experiments, donkey anti-rabbit and sheep anti-mouse HRP-conjugated secondary antibodies (GE Healthcare) were used, diluted 1:5000.

### Immunoblotting

Proteins were separated by 12% SDS-PAGE and transferred to a PVDF membrane (Hybond-P; Amersham Biosciences). Membranes were then blocked with 5% (w/v) not-fat dry milk in PBS, washed three times with PBS containing 0.1% (v/v) Tween 20 (PBST) and incubated overnight at 4°C with primary antibody diluted in PBST containing 1% (w/v) milk. After four washes with PBST, membranes were incubated with HRP-conjugated secondary antibody diluted in PBST for 1 h at room temperature (RT). Detection of the immunocomplexes was performed by enhanced chemiluminescent-based system (SuperSignal West Pico Chemiluminescent Substrate; Pierce).

### Triton X-114 phase separation and Triton X-100 extraction

Triton X-114 phase separation was performed as described [26]. Briefly, at 48 h post-transfection with *Gg NEU3*-Myc, *Gg NEU3-Del*-Myc or *Mm NEU3*-HA, COS7 cells were chilled at 4°C. After three washes with ice-cold PBS, cells were scraped and pelleted. Cells were then resuspended in lysis buffer (10 mM Tris/HCl pH 7.5 containing protease inhibitors), lysed by sonication, clarified by centrifugation at 800 ***g ***for 10 min at 4°C, and the resulting supernatants were diluted in the same buffer to a protein concentration corresponding to 2.0 mg/ml. Samples (100 μl) were treated by adding the same volume of 2% (v/v) precondensed Triton X-114 followed by incubation for 1 h at 4°C and further processed as described in [26]. The detergent and aqueous phases were separated and adjusted to the same final volume and Gg NEU3-Myc, Gg NEU3-Del-Myc and Mm NEU3-HA repartition, together with endogenous protein markers TfR and Cav-1, was analyzed by immunoblotting. Triton X-100 extraction at 4°C was performed to isolate detergent-insoluble membrane proteins [25]. COS7 cells transfected with *Gg NEU3*-Myc and *Gg NEU3-Del*-Myc were collected and sonicated as described above and the clarified supernatants were extracted with 1% Triton X-100 for 30 min at 4°C. After extraction samples were centrifuged at 100000 ***g ***for 1 h at 4°C and pellets, containing the detergent-insoluble membrane proteins, were resuspended in the same volume as supernatants. Gg NEU3-Myc and Gg NEU3-Del-Myc distribution, together with Cav-1 and TfR as markers, was then analyzed by immunoblotting.

### Indirect immunofluorescence analysis

Indirect immunofluorescence experiments were performed on COS7 cells seeded onto glass coverslips and transfected with *Gg NEU3*-Myc or *Gg NEU3-Del*-Myc as described. At 36 h post- transfection cells were fixed with 3% (w/v) paraformaldehyde in PBS for 15 min at RT and treated as described in [26]. Paraformaldehyde was quenched incubating samples with 50 mM NH_4_Cl in PBS for 15 min. After three washes with PBS, cells were permeabilized with 0.3% saponin in PBS (PBS/Sap) for 30 min and then incubated with primary antibodies diluted in PBS/Sap for 1 h.

Subsequently, cells were washed three times with PBS/Sap and incubated with secondary antibodies diluted in PBS/Sap for the same period. Finally, after three washes with PBS/Sap followed by three washes with PBS, specimens were mounted using DakoCytomation Fluorescent Mounting Medium (DakoCytomation) mixed with DAPI (DAPI diluited to 5 μg/ml in Dako medium). Specimens were then analyzed with confocal system LSM-510 META (Carl Zeiss).

## Abbreviations

EST: Expressed Sequence Tag; DMSO: dimethyl sulfoxide; DOP-PCR: degenerate oligonucleotide primed PCR; GRAVY: Grand average of hydropathicity; DANA: 3-acetamido-4-hydroxy-2-(1,2,3- trihydroxypropyl)-3,4-dihydro-2H-pyran-6-carboxylic acid; FBS: fetal bovine serum; 4MU-NeuAc: 4-methylumbelliferyl-α-D-N-acetyl-neuraminic acid; PBS: phosphate buffered saline; EEA1: Early Endosomal Antigen 1; TfR: transferrin receptor; HA: human influenza hemagglutinin; Cav-1: caveolin-1; HRP: horseradish peroxidase.

## Authors' contributions

EG proposed and performed the experimental analysis, interpreted the results and drafted the manuscript; SB participated in the design of the study and the critical evaluation of the results; AP and BV participated in design of the study; EM coordinated the design and the analysis of the protein structural data; GB carried out the genome mining studies; RB was responsible for biochemical and cell microscopy analyses; EM, GB, and RB also participated in the design of the study and the preparation of the final manuscript. All the authors read and approved the final manuscript.
